# Beyond work-hour restrictions: a qualitative study of residents’ subjective workload

**DOI:** 10.1007/s40037-015-0198-4

**Published:** 2015-07-31

**Authors:** Hiroshi Nishigori, Gautam A. Deshpande, Haruo Obara, Osamu Takahashi, Jamiu Busari, Tim Dornan

**Affiliations:** 1 Center for Medical Education, Kyoto University, Yoshida-konoe- cho, Sakyo-ku, 606–8501 Kyoto, Japan; 2 Center for Clinical Epidemiology, St. Luke’s International Hospital, Tokyo, Japan; 3 Department of General Internal Medicine, Okinawa Chubu Hospital, Okinawa, Japan; 4 Educational Development and Research Department, Faculty of Health, Medicine and Life Sciences, Maastricht University, Maastricht, The Netherland

**Keywords:** Subjective workload, Work-hour restrictions, Professionalism

## Abstract

**Introduction:**

Following the introduction of work-hour restrictions, residents’ workload has become an important theme in postgraduate training. The efficacy of restrictions on workload, however, remains controversial, as most research has only examined objective workload. The purpose of this study was to explore the less clearly understood component of subjective workload and, in particular, the factors that influenced residents’ subjective workload.

**Method:**

This study was conducted in Japan at three community teaching hospitals. We recruited a convenience sample of 31 junior residents in seven focus groups at the three sites. Audio-recorded and transcribed data were read iteratively and analyzed thematically, identifying, analyzing and reporting themes within the data and developing an interpretive synthesis of the topic.

**Results:**

Seven factors influenced residents’ subjective workload: (1) interaction within the professional community, (2) feedback from patients, (3) being in control, (4) professional development, (5) private life, (6) interest and (7) protected free time.

**Discussion and conclusion:**

Our findings indicate that residents who have good interaction with colleagues and patients, are competent enough to control their work, experience personal development through working, have greater interest in their work, and have fulfilling private lives will have the least subjective workload.

## Essentials

1. Workload does not equate with duty hours.
2. Good social interactions and being in control make work feel less onerous.
3. Having a good personal life makes it easier to work hard.
4. Careful attention to the social conditions in which residents work helps get the job done.

## Introduction

Residents’ workload has rapidly become one of the most important themes in postgraduate training around the world. Behind this trend lie a number of ethical and legal considerations that have spurred the introduction of work-hour restrictions in many countries. In the United States, the Accreditation Council for Graduate Medical Education (ACGME) developed work-hour guidelines in 2002, revising them in 2011, which limit resident work hours to 80 h per week (further limiting first-year residents to ≤ 16 continuous hours) after the unfortunate and much-publicized Zion case [1, 2]. In the UK, the European Working Time Directive (EWTD) was incorporated into British law in 1998 and a restriction on doctors’ work hours was implemented in 2009, mandating reduction to 48 h per week [1–3].

Since these regulations were implemented, numerous studies have sought to assess their impact on the health care system and postgraduate educational environment. Their impact on health and safety outcomes remains controversial. Proponents argue that strict regulations help to protect patients from dangerously fatigued trainees and protect trainees from dangerously long working hours [3–5]. Opponents argue that multiple handoffs, use of mid-level providers, and decreased time for education detract from residents’ educational experiences, posing a threat to patient safety [6, 7].

Interestingly, much of the previous literature on duty hours has assumed that resident workload is a measurable construct; this is questionable as different residents may experience the same workload in different ways. For example, a diligent and confident resident may thrive in a more demanding environment, which a resident who is work-shy, less confident, or less motivated might find intolerable. Subjective workload may be just as important as objective workload and a better understanding of it could open the way to improving the conditions in which residents learn [8]. However, little is known about the factors that influence residents’ subjective workload, for which further study has been advocated [9].

In order to help medical educators better approach resident workload, we set out to identify factors that contribute to subjective workload, asking ‘What influences residents’ subjective workload?’

## Methods

### Setting

This study was conducted in Japan at three community teaching hospitals active in postgraduate training. Japan is an appropriate place to conduct this research because, despite having an economically advanced health care infrastructure similar to its Western counterparts, work-hour restrictions are not strictly standardized or applied, and data can therefore be obtained from a wide variety of trainees and settings [10, 11].

Three hospitals (A, B, and C) were chosen to participate because they have national reputations for excellence in both clinical practice and education [12], and have shown interest in international (mainly American) training standards, including residents’ duty hours [13]. All sites host competitive junior residency (first and second postgraduate year) training programmes, which have been running for more than a decade.

#### Site A

Site A is a large, 520-bed community hospital offering tertiary care in urban Tokyo. It treats approximately 2000 outpatients a day and is staffed by approximately 130 attending physicians. Forty junior residents (20 first years, 20 s years) participate in a wide variety of clinical rotations. First and second-year residents are primarily responsible for inpatient care, with some outpatient and ambulatory medicine opportunities while on specific rotations. Most rotations include daily rounds and weekly educational conferences in addition to patient care responsibilities; second-year residents have a research requirement as well. Overnight call schedules vary between departments.

#### Site B

Site B is a public, 550-bed tertiary care teaching hospital with a large and active emergency department in central Okinawa. It is staffed by approximately 110 attending physicians and 40 senior residents and employs 56 junior residents (28 first years, 28 s years) who rotate in all major departments. Most rotations include overnight on-call shifts in addition to daily patient care activities such as work rounds and outpatient clinics. During the Emergency Medicine rotation, first-year residents have two-week night floats but do not do overnight on-call during the rest of their rotation. Residents are required to attend daily clinical conferences.

#### Site C

Site C is a 550-bed community hospital located on the western edge of Sapporo, staffed by approximately 100 full-time attending physicians. It employs 39 junior residents (19 first years, 20 s years), who spend approximately 2 months per year in outpatient or emergency room settings, the remainder being devoted primarily to inpatient care. Formal education is focused around a one-hour morning conference held each weekday, which is supplemented by teaching rounds, attending rounds, and other conferences that vary by rotation.

## Participants and educational context

In March 2010, we recruited convenience samples of eight (6 male and 2 female, 5 first-year and 3 second-year) junior residents in two focus groups at Site A, eight (7 male and 1 female, 4 first-year and 4 second-year) junior residents in two focus groups at Site B, and 15 (9 male and 4 female, 8 first-year and 5 second-year; 2 male first-year residents attended two focus groups) junior residents in three focus groups at Site C. Focus groups are a well-established qualitative method, which is especially useful for eliciting trainees’ perspectives [14]. We elected to conduct focus groups rather than individual interviews because a group dynamic helps explore what participants think and why [14]. All focus groups were conducted by the first (HN) and third author (HO) in conference rooms without any supervising physicians being present and lasted approximately 45–90 min.

Ethical approval was granted by Institutional Review Board or Residency Council of the participating institutions, as appropriate. Participation was voluntary and all participants gave written, informed consent.

## Analysis

All discussions were audio-recorded and transcribed verbatim. Data were read iteratively by HN and analyzed thematically, identifying, analyzing and reporting patterns (themes) within the data and developing an interpretive synthesis of the topic [15]. HO read the transcripts separately and discussed the identified themes with HN. This process was adopted to achieve richer interpretation of data. As far as possible, participants’ own words are used to illustrate the main themes and show how social influences could make the same observable amount of work a heavier or lighter subjective workload.

## Results

Seven factors influencing residents’ subjective workload are illustrated below and in Table 1 with excerpts from representative transcripts.

**Table 1:** Elements influencing residents’ subjective workload

(1) Interaction within the professional community
(2) Feedback from patients
(3) Being in control
(4) Professional development
(5) Private life
(6) Interest
(7) Protected free time

1. Interaction within the professional community

Given equal responsibility and objective workload, workload felt heavier when respondents were isolated and lighter when they had meaningful or supportive interactions with peers and colleagues.

Even when [the schedule] is hectic, I enjoy it very much as long as I am in sync with my environment. If I get along well with those around me, whether it is my physician-mentor or the nurses, I really enjoy being busy. But if I can’t get along with the whole flow or can’t communicate well, the same level of work overwhelms me and it just turns into pressure. (Resident from Site A)

I feel stressed when I’m struggling alone, for example, when there are no senior colleagues with whom I can talk things over. Even though they may not help you in a practical sense, they may give you a bit of useful advice. You at least feel much better and may even willingly accept a busy situation. (Resident from Site C)

2. Feedback from patients

Appreciation from patients also affected participants’ perceptions of their workload. Regardless of how busy participants were, patients’ expressions of gratitude improved negative feelings. Complaints from patients about participants’ work, in contrast, made them feel negative about their workload. A similar negative effect was identified when patients failed to respond to participants’ work:

I feel happy and rewarded when patient say things like, ‘Thank you doctor for saving me in the emergency department’. That kind of thing makes my day. But, on the other hand, I want to cry when a patient complains…When I failed to insert an IV cannula and the patient told me to ‘Go away and don’t come back’. I thought, ‘Oh gosh, I am here doing my best at 2 in the morning’. (Resident from Site A)

3. Being in control

Participants entering junior residency were still novices to medical practice despite having completed their undergraduate medical education curriculum and were therefore limited in their ability to make independent decisions. They were not able to control their work because they had to consult with their supervisors about their clinical decision-making. Therefore, their work depended on the schedule and availability of their supervisors, which was reported as a stressor. As participants advanced in their training and became more competent, they were better able to manage patients independently and control their schedule, which made them feel less stressed.

For example, you want to consult with your senior, but he can’t find time now. You wait, but it pushes other things you need to do later and later. But once you are in the second year, you start to know more and be confident enough to do things on your own judgment, to a degree. You start to recognize when you need a consult versus what decisions you can make on your own. (Resident from Site C)

4. Professional development

When participants felt that their work was contributing to their professional development, their subjective workload decreased. When they were asked to do tasks that contributed less to their professional development, or to do tasks that could easily be done by others, they felt negative about their workload.

Even if I couldn’t sleep much on night duty or something, if I feel like I’m learning something or …progressing, it’s OK, I can take it. But if I feel like I’m not learning anything, for example, if I had to do a lot of scut work, like hauling equipment or making photocopies, or…something that doesn’t need an MD to do…I feel (stressed)… (Resident from Site A)

5. Private life

Residents’ private life influenced their perceptions of workload; those that felt more stressed in their private life also felt more pressure at work.

What I’m saying is this: It’s not that my private life is under pressure because of the work, it’s the other way around, meaning that I feel more pressured at work because my private life at the moment is a bit complicated. (Resident from Site C)

6. Interest

Unsurprisingly, the residents in our study felt more positive about their work when they were doing tasks in which they were interested and less positive when they had little interest in their tasks, regardless of its contribution to their professional development.

When there is an emergency patient or, like, a patient with meningitis, I want to be called and in fact I used to show up whether it was midnight or one in the morning. My motivation goes up when there’s a match between the area I’m interested in and the work that I do. (Resident from Site C)

7. Protected free time

Participants felt they could manage their heavy workload if they had a day off. Protected free time reduced their subjective workload.

I had strongly wanted to have a day off either on Saturday or Sunday for a long time. If we had one of those days off, I would not mind working until midnight on weekdays. I think I could manage (working for long hours). (Resident from Site B)

## Discussion and conclusion

### Principal findings and meanings

Using a qualitative approach to workload, this study goes beyond counting duty hours [9] to identify the critical elements that influence resident workload. We identified that quality of residents’ interactions with colleagues and patients was a key factor influencing subjective workload. As relative novices, they needed not only feedback from fellow professionals, but also the reciprocal support that came from interacting with patients.

Residents’ competence was another important element in improving subjective workload, helping them feel in control of their work as well as work in a time-efficient way. Similar to this was residents’ quality of learning; when trainees felt their work was contributing to their professional development, it weighed less heavily on them. Interest in their clinical obligations motivated them.

Finally, the quality of residents’ lives outside work influenced their subjective workload. The term ‘work-life balance’ has been increasingly used in the context of changing priorities in health care careers. Previous arguments have focused on a simple dynamic: as time is limited, doctors often must choose between work at the expense of other facets of their lives. Our findings suggest, in contrast, that there is a complicated and dynamic interplay between the two, in which one actively enriches the other.

In sum, our findings indicate that residents who have good interaction with colleagues and patients, are competent enough to control their work, experience personal development through working, have greater interest in their work, and have fulfilling private lives will have the least subjective workload.

### Relationship to other publications

Other studies have linked residents’ working conditions with burnout and stress [16]. Nyssen et al. reported that lack of control over time management was a source of stress [17]. While this remains true, our study adds the fact that workload itself may have the capacity to either enrich residents’ lives, or leave them feeling dissatisfied and stressed. Our study does so by adopting the relatively neutral concept of workload, rather than burnout or stress, as a conceptual orientation. Previous authors have recommended the term ‘pro-social behaviour’, referring to acts of assistance that ‘produce positive benefits for both the recipient and the helper’, in preference to altruism, which entails self-sacrifice [18, 19]. Self-sacrifice may also be beneficial, but is likely less sustainable in the long run. The benefit to our participants of positive feedback (from both colleagues and patients) illustrates the effect of prosocial behaviour on improving subjective workload. Though perhaps counterintuitive, heavy loads, as long as they entail prosocial behaviours, may actually be beneficial to trainee physicians.

### Strengths and limitations

The strength of this research lies in its applications to residency curriculum reform, especially in understaffed situations in which work-hour restrictions are untenable. This study takes the concept of workload, which is of great concern to the medical education community and heretofore primarily seen negatively in the context of medical training, and recasts it in a more balanced light, showing how organizations and individuals could be helped to benefit without loss of productivity. Its main limitation is that it was conducted in a single culture, which has traditionally viewed self-sacrifice as a positive value under the influence of Bushido [20]. The transferability of our findings beyond the Japanese context cannot be guaranteed, though it defines a topic for further research in other contexts and cultures. Meantime, the research provides educational leaders and residents themselves with ideas as to how they could structure programmes and engage in work and life in a way that fosters their clinical performance and well-being.
